# Comparison of Health, Quality of Life, and Psychological and Cognitive Function Between Perimenopausal and Postmenopausal Women: A Cross-Sectional Study

**DOI:** 10.3390/healthcare14121770

**Published:** 2026-06-19

**Authors:** Jawahr Alagil, Alaa M. Albishi

**Affiliations:** Rehabilitation Health Sciences Department, College of Applied Medical Sciences, King Saud University, Riyadh 11433, Saudi Arabia; aalbeshi@ksu.edu.sa

**Keywords:** menopause, perimenopause, post-menopause, cognitive function, quality of life, psychological health, Saudi women

## Abstract

**Background:** Menopause is associated with hormonal changes that may influence cognitive function, psychological health, and quality of life, but data on Middle Eastern populations remain scarce. **Methods:** A cross-sectional study was conducted among 220 Saudi women (110 perimenopausal, 110 postmenopausal) in Riyadh. Cognitive function was assessed with the MMSE-2; quality of life with SF-36 and MENQOL; and psychological distress with PHQ-4 and PSS-10. Group comparisons used the Mann–Whitney U test; associations with Spearman’s correlation; and multivariable logistic regression adjusted for age, BMI, education, and anxiety. **Results:** In unadjusted analyses, perimenopausal women had higher MMSE-2 scores (median 30 vs. 29, *p* = 0.002, r = 0.211). Postmenopausal women reported greater vasomotor symptoms (*p* < 0.001, r = 0.090) but better emotional well-being (*p* = 0.038, r = 0.140). After adjustment for age, menopausal status was not a significant predictor of lower cognitive function (OR = 1.28, 95% CI: 0.56–2.92, *p* = 0.560). Age was the only significant predictor (OR = 1.10, 95% CI: 1.03–1.17, *p* = 0.003). **Conclusions:** The unadjusted difference in MMSE-2 scores between perimenopausal and postmenopausal women was small and not independent of age. Age, not menopausal status, was the primary factor associated with cognitive performance. Preventive strategies should target modifiable factors such as physical activity and vasomotor symptom management. Longitudinal studies with domain-specific cognitive tests are needed.

## 1. Introduction

The World Health Organization (WHO)’s Scientific Group on Research in the Menopause defines natural menopause as the permanent cessation of menstruation due to the loss of ovarian follicular activity. Standardised terminology distinguishes premenopause, perimenopause, the menopausal transition, and postmenopause based on their timing relative to the final menstrual period (FMP) [1,2]. Menopause is frequently associated with vasomotor, musculoskeletal, and fatigue-related symptoms that may limit physical activity and social participation. The biological changes accompanying menopause have been linked to an increased risk of dementia in women [3] and to cardiovascular disease, which has become more prevalent in older women, approaching rates observed in men despite a lower incidence earlier in life [4]. Health outcomes during menopause are heterogeneous and may be influenced by modifiable factors, including physical activity, social engagement, body weight, and educational attainment [5]. Early menopause is associated with poorer cognitive performance and a higher burden of depressive symptoms compared to later menopause [6]. Depressive symptoms during menopause often result from interactions between hormonal changes, particularly declining oestrogen levels, and psychosocial stressors such as caregiving responsibilities and evolving social roles [7]. Depression during menopause significantly impairs quality of life across physical, psychological, social, and sexual domains [8].

Research from Saudi Arabia underscores the influence of cultural context on menopausal experiences. A recent qualitative study among Saudi women found that menopause is often regarded as a natural life stage, but symptoms are frequently under-reported due to cultural norms [9,10]. Population-based data indicate a high prevalence of menopausal symptoms, with musculoskeletal pain, fatigue, and vasomotor symptoms being most common, and severity varying by menopausal stage [11]. Nevertheless, cognitive and mental health outcomes related to menopause remain underrepresented, constituting only about 2% of menopause-focused neuroimaging studies published between 1995 and 2017 [12]. Cultural conservatism and limited access to mental health services may further impede recognition of cognitive and affective symptoms among postmenopausal Saudi women [13].

Given these gaps, we conducted a cross-sectional study to compare cognitive function, psychological health, and quality of life between perimenopausal and postmenopausal Saudi women. We hypothesized that postmenopausal women would exhibit lower cognitive scores and a higher burden of menopausal symptoms compared to perimenopausal women and that cognitive function would be negatively associated with psychological distress and menopausal symptom severity. The findings are intended to inform preventive strategies tailored to the Saudi cultural context. The MMSE-2 is a global screening tool for possible cognitive impairment (clinical threshold), not for subtle domain-specific changes. We therefore also examine it as a continuous measure.

## 2. Methods

### 2.1. Study Design

A cross-sectional study was conducted in Riyadh, Saudi Arabia [14]. A convenience sampling method was used to recruit participants from two main settings: (1) primary health care centers in Riyadh, and (2) the King Salman Community Centre and King Saud University.

### 2.2. Participants and Eligibility Criteria

Participants were eligible if they were Saudi women aged 40 years or older; able to read, understand, and communicate in Arabic; residents of Riyadh; and in self-reported good general health, defined as the absence of any serious or uncontrolled medical, neurological, or psychiatric condition that would independently impact cognitive or physical function. Exclusion criteria included a history of diagnosed neurological disorders (e.g., Parkinson’s disease, dementia), major psychiatric disorders (e.g., schizophrenia, bipolar disorder), or uncontrolled chronic medical conditions (e.g., advanced heart disease, cancer undergoing active treatment).

### 2.3. Sample Size and Sampling

The sample size was calculated using Epi Info™ StatCalc (version 7.2) for a cross-sectional study with two independent groups. Input parameters: two-sided confidence level 95%, power 80%, ratio 1:1, estimated prevalence of lower cognitive function (MMSE-2 < 27) in postmenopausal women = 25% and in perimenopausal women = 15%. The required sample size was 306 women (153 per group). Because convenience sampling was used, we aimed to recruit 30% more (398), but practical constraints limited us to 220 participants (110 per group), which still exceeds the minimum requirement. The lower achieved sample is acknowledged as a limitation.

### 2.4. Data Collection Procedures

**Figure 1:**
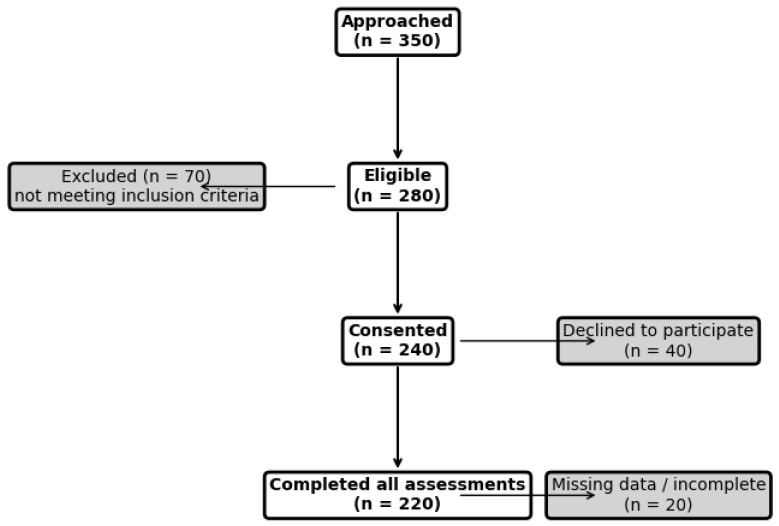
Participant recruitment and data collection flow diagram.

Data were collected during in-person visits by a team of trained research assistants (master’s students in rehabilitation sciences). All assistants underwent uniform training on administering the questionnaires and cognitive assessments to ensure consistency. Written informed consent was obtained from all participants before data collection. All self-report questionnaires were completed in a private room. Participants were assured that they could skip any question, especially on sensitive topics (e.g., MENQOL sexual domain). A participant flow diagram is provided in Figure 1.

### 2.5. Measurement Instruments

All instruments were used in their validated Arabic versions.

#### 2.5.1. Mini-Mental State Examination (MMSE-2)

Cognitive function was assessed using the MMSE-2, a 30-point screening tool evaluating orientation, memory, attention, language, and problem-solving abilities. Higher scores indicate better cognitive function. The Arabic version has demonstrated strong reliability (Cronbach’s α = 0.88) and test–retest reliability (ICC = 0.96) [15]. A score < 27 was used to indicate possible cognitive impairment, based on the Arabic validation by Albanna et al. [16], which recommended this cutoff for this population, adjusted for education (≤12 years). Our sample had higher education, so this cutoff is conservative.

#### 2.5.2. Perceived Stress Scale (PSS-10)

Perceived stress over the past month was measured with the PSS-10, a 10-item scale scored 0–40; higher scores indicate greater stress. The Arabic version has good reliability (α = 0.80; ICC = 0.90) [17].

#### 2.5.3. Patient Health Questionnaire-4 (PHQ-4)

The PHQ-4 is a 4-item ultra-brief screener for anxiety and depression. Each item is scored 0–3, giving total scores 0–12 (normal: 0–2, mild: 3–5, moderate: 6–8, severe: 9–12). The Arabic version has strong psychometric properties [18].

#### 2.5.4. Short Form-36 Health Survey (SF-36)

Health-related quality of life was measured with the Arabic SF-36. The eight domains are scored 0–100, with higher scores indicating better quality of life. The scale has demonstrated strong reliability (α = 0.92; ICC = 0.98) [19].

#### 2.5.5. Menopause-Specific Quality-of-Life Questionnaire (MENQOL)

The MENQOL is a 29-item instrument covering vasomotor, psychosocial, physical, and sexual domains. Each item is scored 1–8; higher scores reflect worse symptom-specific quality of life. The scale has shown good reliability (ICC = 0.70–0.81) and validity in peri- and postmenopausal women [20].

### 2.6. Statistical Analysis

All analyses were performed using IBM SPSS Statistics, Version 27.0.1. Normality was assessed using the Shapiro–Wilk test and visual inspection of Q–Q plots, confirming non-normal distributions; consequently, non-parametric tests were used. Descriptive statistics are presented as frequencies (%), means ± SD, or medians (IQR) as appropriate. Group comparisons were made with the Mann–Whitney U test, and effect sizes (*r*) were calculated. Correlations were assessed using Spearman’s rho. To account for potential confounders, a binary logistic regression was performed with cognitive impairment (MMSE-2 < 27) as the outcome, and menopausal status, age, BMI, and anxiety as predictors. Normality of continuous variables was assessed using both statistical and graphical methods. The Shapiro–Wilk test was applied to assess deviations from normality, and visual inspection using histograms and quantile–quantile (*Q–Q*) plots confirmed the data’s non-normality.

Continuous demographic variables, including age, weight, height, and BMI, are summarised as medians and interquartile ranges (IQRs). Continuous outcome variables, including quality-of-life scores (SF-36), psychological symptoms (PHQ-4 and PSS-10), menopausal symptoms (MENQOL), and cognitive function (MMSE), are also summarised as medians and interquartile ranges (IQRs) because the variables were not normally distributed.

The Mann–Whitney U test was used to compare outcome measures between perimenopausal and postmenopausal women, and effect sizes (r) were calculated to assess the magnitude of group differences. The relationships between MMSE scores and health-related, psychological, and menopausal variables were assessed using Spearman’s rho. To account for potential confounding, a multivariable logistic regression analysis was performed using a binary logistic regression was performed with all a priori confounders entered simultaneously, menopausal status, age, BMI, education, and anxiety, with cognitive decline (yes/no) as the dependent variable and menopausal status, age, and other relevant variables as independent predictors. All tests were two-tailed, with *p* < 0.05 considered statistically significant.

## 3. Results

### 3.1. Demographic and Clinical Characteristics

A total of 220 women participated in this study, with equal representation of perimenopausal (110; 50%) and postmenopausal (110; 50%) participants. The mean age of the sample was 52.35 ± 9.05, with an average body weight of 75.00 ± 16.05 kg, a mean height of 156.98 ± 12.05 cm, and a mean BMI of 32.00 ± 6.43. Regarding educational level, 46.8% held a bachelor’s degree, 15.5% had attained postgraduate education, 15.5% had only completed high school, 9.1% held a diploma, 6.8% had only completed middle school, 2.7% had only attained elementary education, and 3.6% were illiterate. More than half of the participants were employed (55.5%), while 28.6% were unemployed, and 15.9% were retired. Concerning chronic health conditions, 15.9% reported high cholesterol levels, 8.2% reported hypertension, 5.5% reported diabetes, 1.4% reported heart disease, 5.9% reported other chronic illnesses, 25.9% had multiple chronic conditions, and 37.3% reported no chronic diseases. Urinary incontinence was reported by 27.3% of the sample, with 14.1% having received treatment. Pelvic organ prolapse was reported by 17.7% of the participants, and 9.5% had received treatment. Additionally, 62.3% of the women reported that they had visited a gynaecologist (Table 1).

The figure below shows the participant recruitment and data collection flow of 350 women approached, 220 completed all assessments (Figure 1).

### 3.2. Health, Quality of Life, and Psychological and Cognitive Measures

The mean scores for functional and psychological measures were as follows: SF-36 (559.99 ± 136.99), PHQ (2.49 ± 2.30), PSS (14.85 ± 6.08), MENQOL (4.27 ± 1.38), and MMSE (28.41 ± 2.69) (Table 2).

A comparison of health-related quality of life, psychological symptoms, menopausal symptoms, and cognitive function between perimenopausal and postmenopausal women revealed several significant differences. Within the SF-36 subscales, emotional well-being differed significantly between groups (*p* = 0.038), with a small effect size (r = 0.140). Perimenopausal women also reported significantly better pain scores (*p* = 0.009), indicating a small-to-moderate effect (r = 0.175). No significant differences were found in the other SF-36 domains or in the total SF-36 score. Psychological outcomes assessed with the PHQ-4 showed no significant differences in anxiety or depression, with negligible to small effect sizes (r ≤ 0.115). Perceived stress (PSS) also did not differ significantly between groups (*p* = 0.097), with a small effect (r = 0.112). Among the MENQOL domains, only the vasomotor domain showed a significant difference (*p* < 0.001), with a small effect size (r = 0.090), indicating more pronounced vasomotor symptoms among postmenopausal women; the psychosocial, physical, sexual, and total MENQOL scores showed no significant differences. Concerning cognitive outcomes, perimenopausal women had significantly higher MMSE scores in unadjusted analyses (*p* = 0.002), with a small effect size (r = 0.211). However, both groups demonstrated median scores close to the maximum value, suggesting a potential ceiling effect and limited clinical relevance of this difference. Furthermore, after adjusting for age, this association was no longer independent.

A logistic regression analysis was performed to evaluate whether menopausal status, age, and BMI were independently associated with cognitive decline. Binary logistic regression was performed with all a priori confounders entered simultaneously: menopausal status, age, BMI, education, and anxiety. Age was retained as a significant predictor of cognitive decline (*p* = 0.002), whereas BMI and menopausal status were not included in the final model. These findings indicate that age, rather than menopausal status or BMI, was independently associated with cognitive decline.

### 3.3. Relationship Between Cognition and Quality of Life

Correlation analyses examined the relationship between cognitive function, measured by the Mini-Mental State Examination (MMSE), and multiple health-related, psychological, and menopausal variables (Table 3). MMSE showed significant positive correlations with role limitations due to physical health (r = 0.171, *p* = 0.011) and pain (r = 0.136, *p* = 0.044), as well as a borderline significant correlation with the SF-36 total score (r = 0.132, *p* = 0.050), indicating that better cognitive performance was associated with fewer physical limitations and more favourable pain and overall functioning. No significant correlations were found with other SF-36 domains, including physical functioning, emotional well-being, social functioning, energy/fatigue, and general health. Regarding psychological measures, MMSE demonstrated a significant negative correlation with anxiety (r = −0.154, *p* = 0.022), suggesting that higher anxiety levels were associated with lower cognitive performance, while depression, total PHQ-4 scores, and perceived stress (PSS-10) showed no significant associations. For menopausal symptoms, MMSE was significantly negatively correlated with the vasomotor (r = −0.197, *p* = 0.003) and psychosocial (r = −0.173, *p* = 0.010) domains of MENQOL, indicating that more severe vasomotor and psychosocial symptoms were linked to poorer cognitive function, while the physical, sexual, and total MENQOL scores were not significantly related to MMSE. Overall, the findings indicate that specific aspects of physical functioning, anxiety, and menopausal symptom burden show meaningful associations with cognitive performance among the participants (Table 3).

### 3.4. Multivariate Analysis of Factors Associated with Cognitive Function

To account for potential confounders, a binary logistic regression was performed with lower cognitive function (MMSE-2 < 27) as the outcome. The final model included menopausal status, age, BMI, education, and anxiety. As shown in Table 4, after adjustment, age was the only significant predictor (OR = 1.10, 95% CI: 1.03–1.17, *p* = 0.003). Menopausal status was not independently associated with cognitive function (OR = 1.28, 95% CI: 0.56–2.92, *p* = 0.560). The model explained 12% of the variance (Nagelkerke R^2^ = 0.12). A summary of the key unadjusted findings is presented in Table 5.

## 4. Discussion

We investigated associations between menopausal status, cognitive function, psychological health, and quality of life. Our results show that perimenopausal women displayed higher MMSE-2 scores (median 30 vs. 29, *p* = 0.002) and lower vasomotor symptom burden (*p* < 0.001) than postmenopausal women, but postmenopausal women reported better emotional well-being (*p* = 0.038). After adjusting for age, the association between menopausal status and cognitive function was no longer statistically significant (Table 4), indicating that age is the primary driver of the observed unadjusted differences.

### 4.1. Health, Quality of Life, and Psychological and Cognitive Function Between Perimenopausal and Postmenopausal Women

In terms of physical health and quality of life, perimenopausal women exhibited significantly better physical functioning and reported lower pain scores compared to postmenopausal women. These results align with previous research indicating that oestrogen decline after menopause is associated with reductions in muscle mass, which in turn may decrease functional capacity and increase musculoskeletal discomfort [21,22,23], bone density, and connective tissue elasticity, which, in turn, decrease functional capacity and increase musculoskeletal discomfort [22,23]. The effect sizes observed in this study suggest that these differences may be clinically meaningful and could impact independence and daily functioning. Meanwhile, postmenopausal women reported better emotional well-being, consistent with evidence that emotional symptoms often peak during perimenopause and subsequently stabilise or improve in postmenopause as women psychologically adapt to hormonal changes and life transitions [24,25]. Such adaptation may reduce the emotional impact of persistent physical symptoms.

Regarding menopause-specific symptoms, postmenopausal women reported significantly more severe vasomotor symptoms, consistent with the established role of oestrogen deficiency in thermoregulatory instability [26]. Vasomotor symptoms are closely linked to impaired sleep, fatigue, and diminished quality of life [27]. The lack of significant differences in other MENQOL domains indicates that menopause-related quality-of-life impairments are symptom-specific, highlighting the need for targeted symptom management strategies.

On the other hand, the reduced cognitive performance observed among postmenopausal women aligns with studies linking menopause-related hormonal changes to alterations in cerebral blood flow, synaptic plasticity, and neurotransmitter regulation [28,29]. Although the average cognitive scores remained within normal limits, the significant group difference indicates increased vulnerability during the postmenopausal period. Interestingly, regular physical activity has been demonstrated to mitigate both cognitive and physical decline, supporting its role as a key preventive strategy during midlife [30]. The observed association between cognitive performance and physical functioning in this study further underscores the interrelationship between physical and cognitive health.

#### Collinearity Between Age and Menopausal Status

Age and menopausal status are highly correlated, as postmenopausal women are generally older. When both variables are entered into the same regression model, age accounts for most of the variance in cognitive performance. This explains why menopausal status lost significance after age adjustment. The cross-sectional design cannot separate the effects of ageing from those of hormonal transition. Therefore, our findings should be interpreted as showing that age, rather than menopausal status per se, is the more robust predictor of lower MMSE-2 scores in this sample.

Additionally, in terms of psychological health and stress, no significant differences were identified between groups in terms of anxiety, depression, or perceived stress. This finding is consistent with the literature, indicating that psychological distress during midlife is influenced more by psychosocial factors such as coping skills, socioeconomic status, and social support than by menopausal status alone [30,31,32]. The relatively low symptom scores suggest that the study population is psychologically resilient.

## 5. Associations Among Cognitive Function, Quality of Life, Psychological Factors, and Menopause-Related Symptoms

A modest but statistically significant association between cognitive performance and reduced physical-health-related role limitations and lower pain levels. Beyond group comparisons, correlation analyses revealed several significant associations between cognitive performance and specific health-related, psychological, and menopause-related domains.

Cognitive function was positively associated with reduced role limitations attributable to physical health (r = 0.171, *p* = 0.011) and with lower pain levels (r = 0.136, *p* = 0.044). A borderline association was observed with the overall SF-36 total score (r = 0.132, *p* = 0.051). These findings indicate that better cognitive performance is linked to fewer physical restrictions and improved overall functioning. These results are consistent with previous research in which a bidirectional relationship was found between physical and cognitive health in midlife and in older adults, where physical limitations, chronic pain, and reduced mobility may negatively affect cognitive efficiency and engagement in stimulating activities [30,32,33,34]. Evidence from the Study of Women’s Health Across the Nation (SWAN) further supports the interaction between menopausal transition, physical health changes, and cognitive outcomes [22].

Cognitive performance was negatively associated with anxiety, indicating that higher anxiety levels corresponded to lower MMSE scores. Anxiety impairs attentional control, working memory, and executive processing, potentially worsening global cognitive performance [31,34]. In contrast, depression and perceived stress were not significantly correlated with MMSE scores. This lack of association may reflect the relatively low levels of depressive symptoms in this sample or suggest that anxiety-related hyperarousal has a more immediate cognitive impact than general perceived stress.

MMSE scores were negatively correlated with the vasomotor and psychosocial MENQOL domains, indicating that a greater menopause-related symptom burden was associated with poorer cognitive performance. Sleep disturbance linked to vasomotor symptoms is the primary mechanism contributing to cognitive inefficiency, as supported by consistent evidence in the literature [27]. Fatigue resulting from poor sleep further intensifies this effect. Recent neurobiological models describe perimenopause and early postmenopause as a “neurological transition state” in which fluctuating and declining oestrogen levels affect cerebral blood flow, synaptic plasticity, and neurotransmitter regulation [28,29]. Although these vascular and neurotransmitter changes provide an additional explanation, current evidence most suggests sleep disruption as the major contributor to the observed relationship between symptom severity and cognitive function.

Psychosocial menopausal symptoms, including irritability, mood fluctuations, and reduced confidence, may interact with cognitive complaints through stress-related neuroendocrine pathways [24,25]. Although the correlations observed were modest, these findings highlight the multidimensional nature of cognitive health during menopause and suggest that cognitive vulnerability is more closely linked to specific symptom domains than to overall psychological distress. The concept of cognitive reserve emphasises the importance of modifiable factors such as education, occupational complexity, and social engagement in enhancing brain resilience against symptoms and decline. Promoting reserve-building activities may buffer the impact of psychosocial symptoms and support cognitive functioning during the menopausal transition. This perspective allows the identification of actionable strategies for fostering cognitive resilience in middle-aged women.

Collectively, these findings demonstrate that cognitive health in middle-aged women is closely interconnected with physical functioning, anxiety, and menopause-specific symptom burden, highlighting the need for comprehensive assessment and integrative interventions that address physical, psychological, and menopause-related factors simultaneously. Leading frameworks, such as the 2020 Lancet Commission report, also emphasise these priorities by offering a prevention perspective for cognitive health and highlighting the importance of multimodal intervention approaches (for the prevention framework [34]).

Notably, cultural and social factors significantly influence cognitive function and quality of life during menopause and ageing. Social isolation, which may become more prevalent during the menopausal transition, is a well-established risk factor for cognitive decline and diminished psychological well-being. Decreased social engagement has been associated with impairments in memory, executive function, and overall cognitive performance, along with lower quality of life in later years [33,35]. Within the Saudi cultural context, menopausal and older women may face increased social isolation due to shifting family roles, reduced workforce participation, and limited access to age- and gender-appropriate social or physical activity programs. Although family support is generally strong, relying solely on family networks may not provide sufficient cognitive stimulation to prevent age-related cognitive decline [36]. Furthermore, menopause-related symptoms and cognitive concerns are frequently under-discussed, potentially delaying recognition and management [37]. Research focusing on Saudi women of advanced age, particularly in relation to cognitive health and menopause-related quality of life, remains limited. Additionally, there is a lack of culturally tailored preventive and therapeutic programs that address cognitive decline, physical inactivity, and psychosocial well-being among ageing Saudi women. This gap may contribute to unmet healthcare needs and diminished quality of life in this population [38]. These findings underscore the importance of interpreting cognitive and quality-of-life outcomes within a broader sociocultural framework. Addressing biological changes alone is insufficient. Culturally sensitive interventions that promote social engagement, physical activity, and early health education are essential to support cognitive health and enhance quality of life among menopausal and ageing Saudi women.

### Clinical Implications

The findings reveal that menopause is a critical period for preventive intervention. Encouraging regular physical activity, strength training, and balance exercises before and after menopause may help preserve both physical and cognitive function. Furthermore, targeted education on vasomotor symptom management and pelvic floor health should be integrated into women’s health programs to support long-term quality of life.

## 6. Limitations and Future Directions

Several limitations should be acknowledged. First, the cross-sectional design limits causal inference; we cannot determine whether menopausal status directly contributes to cognitive differences or whether other age- or health-related factors explain the observed associations. Longitudinal studies are needed.

Second, cognitive assessment relied on the MMSE-2. As noted by the reviewers, this instrument is subject to a ceiling effect, particularly in a well-educated sample. Although we observed a statistically significant difference in scores between groups, the median scores for both groups (30 and 29) were at or near the maximum possible. This suggests the MMSE-2 may lack sensitivity to detect subtle, domain-specific cognitive changes (e.g., in executive function or processing speed) that are characteristic of the menopausal transition. Future studies should employ a comprehensive neuropsychological battery.

Third, most outcome measures were self-reported, introducing potential recall and social desirability biases. In culturally conservative contexts, psychological and sexual symptoms may be under-reported. Fourth, the sample had relatively high educational attainment, which may reflect increased cognitive reserve, potentially attenuating observable cognitive decline and limiting generalizability to women with lower educational levels or from rural regions. Fifth, potential confounding factors such as hormone therapy use, sleep quality, dietary habits, and objectively measured physical activity levels were not fully controlled and may have influenced outcomes. Finally, the convenience sampling method and sample size may limit statistical power and generalizability.

Future studies should utilise longitudinal designs, incorporate objective hormonal and metabolic biomarkers, and examine the moderating effects of physical activity, social engagement, and health education interventions on cognitive trajectories during and after menopause.

## 7. Conclusions

Perimenopausal women exhibited higher MMSE-2 scores than postmenopausal women, but the difference was small (median 30 vs. 29) and likely influenced by the ceiling effect of the MMSE-2. After adjusting for age, menopausal status was no longer an independent predictor of cognitive function, suggesting that age is the dominant factor. Postmenopausal women reported more vasomotor symptoms but also better emotional well-being, indicating psychosocial adaptation. These findings reinforce the importance of preventive strategies targeting modifiable factors such as physical activity, vasomotor symptom management (including sleep hygiene), and social engagement. Future longitudinal studies should use domain-specific cognitive tests and more diverse sampling (beyond the Riyadh convenience sample with higher education levels) to confirm these results.

## Figures and Tables

**Table 1:** Demographic and clinical characteristics of peri- and postmenopausal women (N = 220).

Variable	Category Age	BMI	N (%)
Group	Perimenopausal 45.32 ± 3.50	28.76 ± 4.78	110 (50%)
	Postmenopausal 59.39 ± 7.23	30.56 ± 4.72	110 (50%)
Educational Level	Bachelor		103 (46.8%)
	Postgraduate		34 (15.5%)
	High school		34 (15.5%)
	Diploma		20 (9.1%)
	Middle school		15 (6.8%)
	Elementary		6 (2.7%)
	Illiterate		8 (3.6%)
Employment Status	Employed		122 (55.5%)
	Retired		35 (15.9%)
	Unemployed		63 (28.6%)
Presence of Chronic Diseases	High cholesterol		35 (15.9%)
	Hypertension		18 (8.2%)
	Diabetes		12 (5.5%)
	Heart disease		3 (1.4%)
	Other		13 (5.9%)
	Multiple chronic diseases		57 (25.9%)
	No chronic disease		82 (37.3%)
Urinary Incontinence			60 (27.3%)
Treatment for Incontinence			31 (14.1%)
Pelvic Organ Prolapse			39 (17.7%)
Treatment for Prolapse			21 (9.5%)
Visit to Gynaecologist			137 (62.3%)

**Table 2:** Comparison of health, quality of life, and psychological and cognitive measures between perimenopausal and postmenopausal women.

Scale/Subscale	Perimenopausal Median (IQR)	Postmenopausal Median (IQR)	Effect Size	*p* Value
**SF-36**				
Physical functioning	82.50 (65.00–95.00)	75.00 (55.00–85.00)	0.212	0.002 *
Role limitations (physical)	100.00 (50.00–100.00)	75.00 (25.00–100.00)	0.102	0.131
Role limitations (emotional)	100.00 (33.33–100.00)	100.00 (33.33–100.00)	0.033	0.620
Energy/fatigue	60.00 (50.00–75.00)	60.00 (50.00–70.00)	0.018	0.791
Emotional well-being	72.00 (64.00–80.00)	76.00 (64.00–88.00)	0.140	0.038 *
Social functioning	75.00 (62.50–100.00)	87.50 (62.50–100.00)	0.090	0.184
Pain	77.50 (60.00–90.00)	67.50 (47.50–80.00)	0.175	0.009 *
General health	70.00 (60.00–80.00)	70.00 (60.00–80.00)	0.025	0.708
Total SF-36	597.75 (472.33–670.00)	593.67 (467.33–661.00)	0.047	0.487
**PHQ-4**				
Anxiety	1.00 (0.00–2.00)	1.00 (0.00–2.00)	0.002	0.980
Depression	1.00 (0.00–2.00)	1.00 (0.00–2.00)	0.115	0.088
Total PHQ-4	2.00 (1.00–4.00)	2.00 (0.00–4.00)	0.078	0.247
PSS	16.00 (12.00–19.00)	15.00 (9.00–19.00)	0.112	0.097
**MENQOL**				
Vasomotor domain	3.00 (1.67–4.00)	3.83 (3.00–5.00)	0.090	<0.001 *
Psychosocial domain	4.00 (3.17–5.17)	4.17 (3.17–5.17)	0.299	0.953
Physical domain	4.17 (3.22–5.11)	4.44 (3.67–5.22)	0.004	0.183
Sexual domain	2.17 (1.00–3.67)	1.00 (1.00–4.33)	0.090	0.931
Total MENQOL	4.17 (3.22–5.11)	4.44 (3.67–5.22)	0.006	0.183
MMSE	30.00 (29.00–30.00)	29.00 (27.00–30.00)	0.211	0.002 *

MMSE—Mini-Mental State Examination score; SF-36—Self-Reported Health Survey; PHQ-4—Patient Health Questionnaire; PSS—Perceived Stress Scale; MENQOL—Menopause-Specific Quality of Life Questionnaire. * *p* < 0.05, significant.

**Table 3:** Spearman correlation between menopause-related symptoms, psychological factors, cognition, and quality of life.

Variable		Correlation Coefficient (r)	*p*-Value
SF-36	Physical function	0.106	0.118
	Role limitations—physical	0.171 *	0.011 *
	Role limitations—emotional	0.079	0.241
	Energy/fatigue	0.095	0.161
	Emotional well-being	0.043	0.528
	Social functioning	0.088	0.192
	Pain	0.136 *	0.044
	General health	0.004	0.948
	SF-36 total score	0.132 *	0.050
PHQ-4	Anxiety	−0.154 *	0.022 *
	Depression	−0.032	0.635
	PHQ-4 total	−0.095	0.160
	PSS-10	−0.023	0.737
MENQOL	Vasomotor domain	−0.197 **	0.003 *
	Psychosocial domain	−0.173 *	0.010 *
	Physical domain	−0.092	0.174
	Sexual domain	0.005	0.938
	MENQOL total score	−0.092	0.174

SF-36—Self-Reported Health Survey; PHQ-4—Patient Health Questionnaire; PSS-10—Perceived Stress Scale; MENQOL—Menopause-Specific Quality of Life Questionnaire. * *p* < 0.05, significant; **, correlation is significant at the 0.01 level.

**Table 4:** Binary logistic regression analysis of factors associated with lower cognitive function (MMSE-2 < 27).

Predictor	B	SE	Wald	*p*-Value	OR	95% CI for OR
**Menopausal status (post vs. peri)**	0.245	0.421	0.34	0.560	1.28	0.56–2.92
**Age (years)**	0.091	0.031	8.62	0.003	1.10	1.03–1.17
**BMI (kg/m^2^)**	0.012	0.021	0.33	0.566	1.01	0.97–1.05
**Education (≤high school vs. ≥diploma)**	−0.512	0.402	1.62	0.203	0.60	0.27–1.32
**Anxiety (PHQ-4 anxiety score)**	−0.078	0.109	0.51	0.475	0.93	0.75–1.15
**Constant**	−5.112	1.502	11.58	<0.001	0.006	

Model fit: Nagelkerke R^2^ = 0.12; Hosmer–Lemeshow χ^2^ = 6.32, *p* = 0.61.

**Table 5:** Summary of main significant associations (unadjusted).

Outcome	Comparison/Association	Effect Size (r or OR)	95% CI	*p*-Value
**MMSE-2 (higher = better)**	Perimenopausal vs. postmenopausal	r = 0.211	–	0.002
**Vasomotor symptoms (MENQOL)**	Postmenopausal vs. perimenopausal	r = 0.090	–	<0.001
**Emotional well-being (SF-36)**	Postmenopausal vs. perimenopausal	r = 0.140	–	0.038
**Pain (SF-36)**	Perimenopausal vs. postmenopausal	r = 0.175	–	0.009
**Lower cognitive function (MMSE < 27)**	Age (per year)	OR = 1.10	1.03–1.17	0.003
**Lower cognitive function**	Menopausal status (post vs. peri) adjusted for age	OR = 1.28	0.56–2.92	0.560

The adjusted model (Table 4) shows that after controlling for age, menopausal status is no longer significant.

## Data Availability

The data supporting the findings of this study are available from the corresponding author upon reasonable request.

## Abbreviations

BMI	Body Mass Index
FMP	Final Menstrual Period
ICC	Intraclass Correlation Coefficient
MENQOL	Menopause-Specific Quality-of-Life Questionnaire
MMSE	Mini-Mental State Examination
MMSE-2	Mini-Mental State Examination, Second Edition
PHQ-4	Patient Health Questionnaire-4
PSS-10	Perceived Stress Scale (10-Item Version)
QoL	Quality of Life
SD	Standard Deviation
SF-36	Short Form-36 Health Survey
SPSS	Statistical Package for the Social Sciences
WHO	World Health Organization

## References

[1] World Health Organization (1996). Research on the Menopause in the 1990s: Report of a WHO Scientific Group.

[2] World Health Organization (1981). Research on the Menopause: Report of a WHO Scientific Group [Meeting held in Geneva from 8 to 12 December 1980].

[3] Ambikairajah A., Walsh E., Tabatabaei-Jafari H., Cherbuin N. (2019). Fat mass changes during menopause: A metaanalysis. Am. J. Obstet. Gynecol..

[4] McAloon C.J., Boylan L.M., Hamborg T., Stallard N., Osman F., Lim P.B., Hayat S.A. (2016). The changing face of cardiovascular disease 2000–2012: An analysis of the world health organisation global health estimates data. Int. J. Cardiol..

[5] Vatankhah H., Khalili P., Vatanparast M., Ayoobi F., Esmaeili-Nadimi A., Jamali Z. (2023). Prevalence of early and late menopause and its determinants in Rafsanjan cohort study. Sci. Rep..

[6] Nakanishi M., Yamasaki S., Stanyon D., Miyashita M., Nakashima T., Miyamoto Y., Ogawa A., Ando S., Nishida A. (2025). Associations among age at menopause, depressive symptoms, and cognitive function. Alzheimer’s Dement..

[7] Santoro N., Roeca C., Peters B.A., Neal-Perry G. (2021). The menopause transition: Signs, symptoms, and management options. J. Clin. Endocrinol. Metab..

[8] Hunter M.S., Chilcot J. (2021). Is cognitive behaviour therapy an effective option for women who have troublesome menopausal symptoms?. Br. J. Health Psychol..

[9] AlSwayied G., Frost R., Hamilton F.L. (2024). Menopause knowledge, attitudes and experiences of women in Saudi Arabia: A qualitative study. BMC Women’s Health.

[10] AlQuaiz A.M., Tayel S.A., Habib F.A. (2013). Assessment of symptoms of menopause and their severity among Saudi women in Riyadh. Ann. Saudi Med..

[11] Kandasamy G., Almaghaslah D., Almanasef M. (2024). A study on anxiety and depression symptoms among menopausal women: A web based cross sectional survey. Front. Public Health.

[12] Abdel-Salam D.M., Mohamed R.A., Alruwaili R.R., Alhablani F.S., Aldaghmi R.M., ALghassab R.E. (2021). Postmenopausal symptoms and their correlates among Saudi women attending different primary health centers. Int. J. Environ. Res. Public Health.

[13] Taylor C.M., Pritschet L., Yu S., Jacobs E.G. (2019). Applying a women’s health lens to the study of the aging brain. Front. Hum. Neurosci..

[14] Von Elm E., Altman D.G., Egger M., Pocock S.J., Gøtzsche P.C., Vandenbroucke J.P. (2007). The Strengthening the Reporting of Observational Studies in Epidemiology (STROBE) statement: Guidelines for reporting observational studies. Lancet.

[15] Albanna M., Yehya A., Khairi A., Dafeeah E., Elhadi A., Rezgui L., Al Kahlout S., Yousif A., Uthman B., Al-Amin H. (2017). Validation and cultural adaptation of the Arabic versions of the Mini–Mental Status Examination–2 and Mini-Cog test. Neuropsychiatr. Dis. Treat..

[16] Cohen S. (1988). Perceived Stress in a Probability Sample of the United States.

[17] Al-Madi A., Al-Emadi S., Al-Hamad A., Harrabi I., Al-Mulla M., Kadhum M. (2012). An Arabic version of the Perceived Stress Scale (PSS-14): Translation and validation in an Arab sample. Soc. Behav. Personal..

[18] Kroenke K., Spitzer R.L., Williams J.B., Löwe B. (2009). An ultra-brief screening scale for anxiety and depression: The PHQ–4. Psychosomatics.

[19] Guermazi M., Allouch C., Yahia M.T.B.A., Huissa T.B.A., Ghorbel S., Damak J., Mrad M.F., Elleuch M.H. (2012). Translation in Arabic, adaptation and validation of the SF-36 Health Survey for use in Tunisia. Ann. Phys. Rehabil. Med..

[20] Hilditch J.R., Lewis J., Peter A., van Maris B., Ross A., Franssen E., Guyatt G.H., Norton P.G. (1996). A menopause-specific quality of life questionnaire: Development and psychometric properties. Maturitas.

[21] El Khoudary S.R., Greendale G., Crawford S.L., Avis N.E., Brooks M.M., Thurston R.C., Karvonen-Gutierrez C., Waetjen L.E., Matthews K. (2019). The menopause transition and women’s health at midlife: A progress report from the Study of Women’s Health Across the Nation (SWAN). Menopause.

[22] Ko S.H., Jung Y.J. (2021). Energy metabolism changes and dysregulated lipid metabolism in postmenopausal women. Nutrients.

[23] Bromberger J.T., Epperson C.N. (2018). Depression during and after the perimenopause: Impact of hormones, genetics, and environmental determinants of disease. Obstet. Gynecol. Clin. N. Am..

[24] Freeman E.W. (2015). Associations of depression with the transition to menopause. Menopause.

[25] Avis N.E., Crawford S.L., Greendale G. (2015). Duration of menopausal vasomotor symptoms over the menopause transition. JAMA Intern. Med..

[26] Khan S.J., Kapoor E., Faubion S.S., Kling J.M. (2023). Vasomotor symptoms during menopause: A practical guide on current treatments and future perspectives. Int. J. Women’s Health.

[27] Brinton R.D., Yao J., Yin F., Mack W.J., Cadenas E. (2015). Perimenopause as a neurological transition state. Nat. Rev. Endocrinol..

[28] Weber M.T., Rubin L.H., Maki P.M. (2014). Cognition in perimenopause. Obstet. Gynecol. Clin..

[29] Erickson K.I., Hillman C., Kramer A.F. (2019). Physical activity, brain, and cognition. Curr. Opin. Behav. Sci..

[30] Avis N.E., Crawford S.L. (2018). Mental health and menopause. Menopause.

[31] Bromberger J.T., Kravitz H.M., Chang Y., Cyranowski J.M., Brown C., Matthews K.A. (2011). Major depression during and after the menopausal transition. Psychol. Med..

[32] Kuiper J.S., Zuidersma M., Oude Voshaar R.C., Zuidema S.U., van den Heuvel E.R., Smidt N. (2015). Social relationships and cognitive decline: A systematic review and meta-analysis. Int. J. Epidemiol..

[33] Evans I.E.M., Martyr A., Collins R., Brayne C., Clare L. (2019). Social isolation and cognitive function in later life: A systematic review and meta-analysis. J. Alzheimer’s Dis..

[34] Eysenck M.W., Derakshan N., Santos R., Calvo M.G. (2007). Anxiety and cognitive performance: Attentional control theory. Emotion.

[35] Almuqbil M., Kraidiye N., Alshmaimri H., Kaabi A.A., Almutiri A., Alanazi A., Hjeij A., Alamri A.S., Alsanie W.F., Alhomrani M. (2022). Postpartum depression and health-related quality of life: A Saudi Arabian perspective. PeerJ.

[36] Abdelaliem S.M.F., Hassan N.M.M., Alqahtani A., Alamer L., Alhomaid N., Alsubaie H., Alsaeed R., Al-Qahtani D., Alenazi M. (2025). Assessing the Relationship Between Depressive Symptoms and Menopausal Quality of Life Among Academic Women in Saudi Arabia. Healthcare.

[37] Hajjar R.R., Atli T., Al-Mandhari Z., Oudrhiri M., Balducci L., Silbermann M. (2013). Prevalence of ageing population in the Middle East and its implications on cancer incidence and care. Ann. Oncol..

[38] Livingston G., Sommerlad A., Orgeta V., Costafreda S.G., Huntley J., Ames D., Ballard C., Banerjee S., Burns A., Cohen-Mansfield J. (2024). Dementia prevention, intervention, and care: 2024 report of the Lancet Standing Commission. Lancet.

